# Special Issue “Adipose Tissue Diseases: Physiopathology, Molecular Mechanism, Diagnosis, and Treatment”

**DOI:** 10.3390/ijms26052068

**Published:** 2025-02-27

**Authors:** Roberto Cannataro, Sandro Michelini, Erika Cione

**Affiliations:** 1 Galascreen Laboratories, University of Calabria, 87036 Rende, Italy; 2 Research Division, Dynamical Business & Science Society, DBSS International SAS, Bogota 110311, Colombia; erika.cione@unical.it; 3 San Giovanni Battista Hospital SMOM, 00148 Rome, Italy; sandromichelini2018@gmail.com; 4 Department of Pharmacy, Health and Nutritional Sciences, University of Calabria, 87036 Rende, Italy

Adipose tissue (AT) is now considered an organ in all respects; therefore, it is customary to refer to it as an adipose organ.

Its role in regulating numerous biochemical processes and metabolic pathways is now well established. AT expresses numerous extra- and intracellular receptors. Therefore, it receives a volume of “messages” from all the organs and tissues that make up the human organism, not only through classic processes like insulin and thyroid hormones, but also through various cytokines; in the same way, as is the case for practically all organs and tissues, AT acts as a secretor of hormones and cytokines. This network is essential for the correct functioning of the human organism. AT, in its normal function, is the primary energy depot, but it influences the districts that use said energy, such as skeletal and cardiac muscle. Therefore, it is in close communicative contact [1,2].

Like all organs and tissues, AT is subject to malfunction induced by an incorrect lifestyle or by real pathological states. This Special Issue aims to take the first step in further underlining the importance of understanding AT’s physiology and pathophysiology.

Obesity is arguably the best-known and studied medical condition, and is a pathology that is certainly multifactorial, with a genetic component but also being strongly influenced by lifestyle, namely, in the form of incorrect eating habits and lack of physical activity [3].

In this sense, non-pharmacological strategies are sought to improve the condition [4]. Wona et al. [5] show how Lycium chinense enhances Glucagon-like peptides, which results in the modulation of obesity and the type II diabetes related to it. Although it is a study on animals, it shows interesting potential for humans.

Obesity and metabolic syndrome are also characterized by a subclinical inflammatory state [6]; to address this issue, Garcia-Garcia et al. [7] propose a combined administration of Arthrospira maxima phycobiliproteins (PBPs), rosiglitazone (RSG), and 17β-estradiol (E), which is evidenced in cell lines through a downregulation of pro-inflammatory and adipogenic genes.

One pathology of the adipose tissue that has gained interest within the scientific community is lipedema, which, until a few years ago, was misdiagnosed as obesity. It is now considered the primary pathology affecting the adipose tissue. A probable genetic cause has been recognized, and its management is not currently pharmacological [8,9]. Understandings of lipedema are still not well known; therefore, the work of Patton et al. [10] marks an exciting moment, as it reports on a group of 360 women affected by lipedema, highlighting comorbidities such as insulin resistance but also a non-negligible inflammatory component. The work of Kempa et al. [11] further demonstrates the interest in this pathology. Kempa et al. analyzed the metabolomics in 25 affected subjects compared to another 25 obese subjects, resulting in an important difference surrounding lipid metabolism.

In some cases, lipedema can affect the functioning of the lymphatic system. Thus, one study in this Special Issue explores the condition of lipo-lymphedema: Weaver et al. [12] show that inflammation of the peri-lymphatic adipose tissue (PLAT) can give rise to dysfunctions of the adipose tissue itself.

There is still much to know about adipose tissue. In the work of Mannelli et al. [13], the activity of a Signal Transducer and Activator of Transcription 3 (STAT3) is highlighted in cancer patients. However, studies in patients with a non-physiological condition could better elucidate these mechanisms, proving decisive in understanding the functioning and regulation of the adipocyte cell cycle.

The manuscripts included in this Special Issue, as well as the scientific literature of the last 15 years, clearly show a strong interest in understanding how AT relates to the rest of the human organism.

In the future, it will be essential to obtain the profiling of the expression of receptors of the adipose cell and secreted cytokines, as well as other mediators such as microRNA. These nucleotide fragments were thought to be found in circulation as by-products, but have since been seen to behave as inter-cellular mediators of protein regulation [14], and are also applicable as possible biomarkers of various physiological and pathological conditions related to adipose tissue, such as type II diabetes [15] or hepatic steatosis [16]. An alteration in miRNA’s profile was also noted in lipedema [17].

Nutrition and physical exercise certainly have a significant impact on AT. Even in a pathological condition like lipedema, the ketogenic diet (KD) is more effective than classic dietary patterns [18]. Therefore, this process should be better analyzed to provide a possible indication of the prevention of the onset of pathology and for its management.

For example, regarding the role of physical exercise, especially resistance training [19], the action of Peroxisome proliferator-activated receptor-gamma coactivator (PGC)-1alpha in the so-called browning of the AT [20] makes the AT itself more metabolically active and, therefore, less prone to the development of obesity.
